# Porencephaly and Psychosis: A Rare Case of Neurological and Psychiatric Intersection

**DOI:** 10.7759/cureus.69486

**Published:** 2024-09-15

**Authors:** Tanya Gupta, Sneha Suresh, Yatika Chadha, Saket S Toshniwal, Ragini Patil

**Affiliations:** 1 Department of Psychiatry, Jawaharlal Nehru Medical College, Datta Meghe Institute of Higher Education and Research, Wardha, IND; 2 Department of General Medicine, Jawaharlal Nehru Medical College, Datta Meghe Institute of Higher Education and Research, Wardha, IND

**Keywords:** encephalomalacia, jerky movements, neurological impairment, porencephaly, psychosis

## Abstract

Porencephaly is an uncommon neurological condition characterized by cystic cavities or holes in the cerebral hemispheres of the brain filled with cerebrospinal fluid. There are two types of porencephaly: acquired porencephaly, also known as pseudo-porencephaly, and congenital porencephaly, also known as true porencephaly.

Acquired porencephaly, also known as encephaloclastic porencephaly, typically results from late prenatal or perinatal vascular lesions caused by arterial ischemic stroke or venous thrombosis. Congenital porencephaly, or genetic porencephaly, arises from maldevelopment during early neuronal migration. Brain lesions associated with congenital porencephaly are thought to result from irregularities in cell migration during development and are often linked to additional brain deformities. Lesions resulting from degenerative disorders caused by vascular, viral, or traumatic events are classified under acquired porencephaly. Familial cases of porencephaly are believed to be caused by mutations in the COL4A1 gene, which lead to brain small-vessel disease with haemorrhage. Due to the variability in lesion size and location, porencephaly presents with a wide range of clinical symptoms.

We report a case of a 41-year-old male who is diagnosed as a case of porencephaly with complaints of withdrawn behaviour, decreased interaction, suspiciousness, delusion of persecution and delusion of reference. These symptoms have started in the past two months. This case report contributes to the growing body of research suggesting a potential link between porencephaly and psychosis, despite the limited available data. Further investigation is required to validate this connection and explore the underlying mechanisms. Continued research into this potential association may help guide future psychosis diagnosis and treatment plans.

## Introduction

A rare condition of the central nervous system called porencephaly is characterized by the presence of degenerative holes in the brain that are filled with cerebrospinal fluid [1]. Numerous writers claim that there are two different kinds of porencephaly: acquired porencephaly, also known as pseudo porencephaly, and true porencephaly, also known as congenital porencephaly [2]. It is caused either by localized brain hemisphere ischemia or, more frequently, by bleeding after birth, and can also result from abnormal prenatal development [1]. Brain lesions known as congenital porencephaly are thought to result from irregularities in cell migration during development and are frequently linked to additional brain deformities [3]. Brain lesions resulting from any degenerative disorder brought on by vascular, viral, or traumatic events are included in acquired porencephaly [2]. It is believed that mutations in the COL4A1 gene, which induce brain small-vessel disease with haemorrhage, are the source of porencephaly in families. Due to the variability in lesion size and location, porencephaly presents with a wide range of clinical symptoms [3]. Psychosis is characterized by abnormalities in perception, thought, emotion, and behaviour. Typical symptoms include delusions, disorganized thinking, hallucinations, and difficulties with social and occupational functioning. Psychosis can result from various neurological and mental disorders, such as bipolar disorder, schizophrenia, and neurodegenerative diseases [1]. However, psychosis in individuals with porencephaly has not been extensively discussed in the medical literature.

Porencephaly is usually associated with cerebral palsy, hemiparesis, epilepsy, mental retardation or other learning disabilities and hemiplegia [4]. Despite generalized brain involvement having stronger evidence of schizophrenia or the initial psychotic episode, specific structural brain abnormalities involving the frontal and temporal lobes have been found in the scientific literature in patients with specific brain involvement [5,6].

## Case presentation

A 41-year-old male presented in the Ear Nose Throat ward of a tertiary care hospital and was admitted with complaints of deliberate self-harm of slashing his neck. Following this, a psychiatry reference was done in view of suicidal ideation. On further evaluation, it was observed that for the last two months, the patient was experiencing withdrawn behaviour which involved decreased interaction with the family members and refusal to step out of his room even on being called multiple times. This was accompanied by irritability and being verbally and physically abusive with family members on trivial things (placement of things at home, in response to being asked to go outside home and meet people) and often without any provocation. He was also noted to be fearful most of the time. When questioned about the above behaviour, the patient reported that he felt as if someone might harm him or kill him and that cameras were installed in his home, through which his thoughts were being controlled. The patient also attributes that the act of self-harm was not his own and that he was made to do the same. The patient was firmly fixed to this belief when given contrary evidence of the same.

Upon examining the mental status of the patient, he was conscious, partially cooperative, and well-oriented to time, place and person and rapport was established with difficulty. The speech was characterized by decreased rate, rhythm, and volume and was not coherent. His mood was subjectively sad but his affect was inappropriate and flaccid (not being able to show expressions and confused). His thought content revealed persecutory delusion (belief in the presence of installed cameras in the house), delusion of reference (belief that people are talking about him, and gets irritated and asks them about the same) and made phenomenon (belief that his thoughts are being controlled and all the people can hear his thoughts). He denied any presence of active death wishes.

On evaluating the patient’s past medical history, it was noted that one year ago, he had episodes of generalized jerky movements of limbs associated with weakness of the right arm and leg, slurring of speech and loss of consciousness. No history of substance use, or trauma, and no history of similar complaints in family members. Socio-occupational impairment is present. No history of previous vascular disease. Magnetic resonance imaging (MRI) revealed encephalomalacia and gliosis in the surrounding area of the left parieto-temporo-occipital region involving the lateral ventricle, leading to the dilatation of lateral, third and fourth ventricle for which venous shunting was done one year ago, following which the patient experienced symptoms of jerky movements, slurred speech, episodes of unconsciousness.

**Figure 1:**
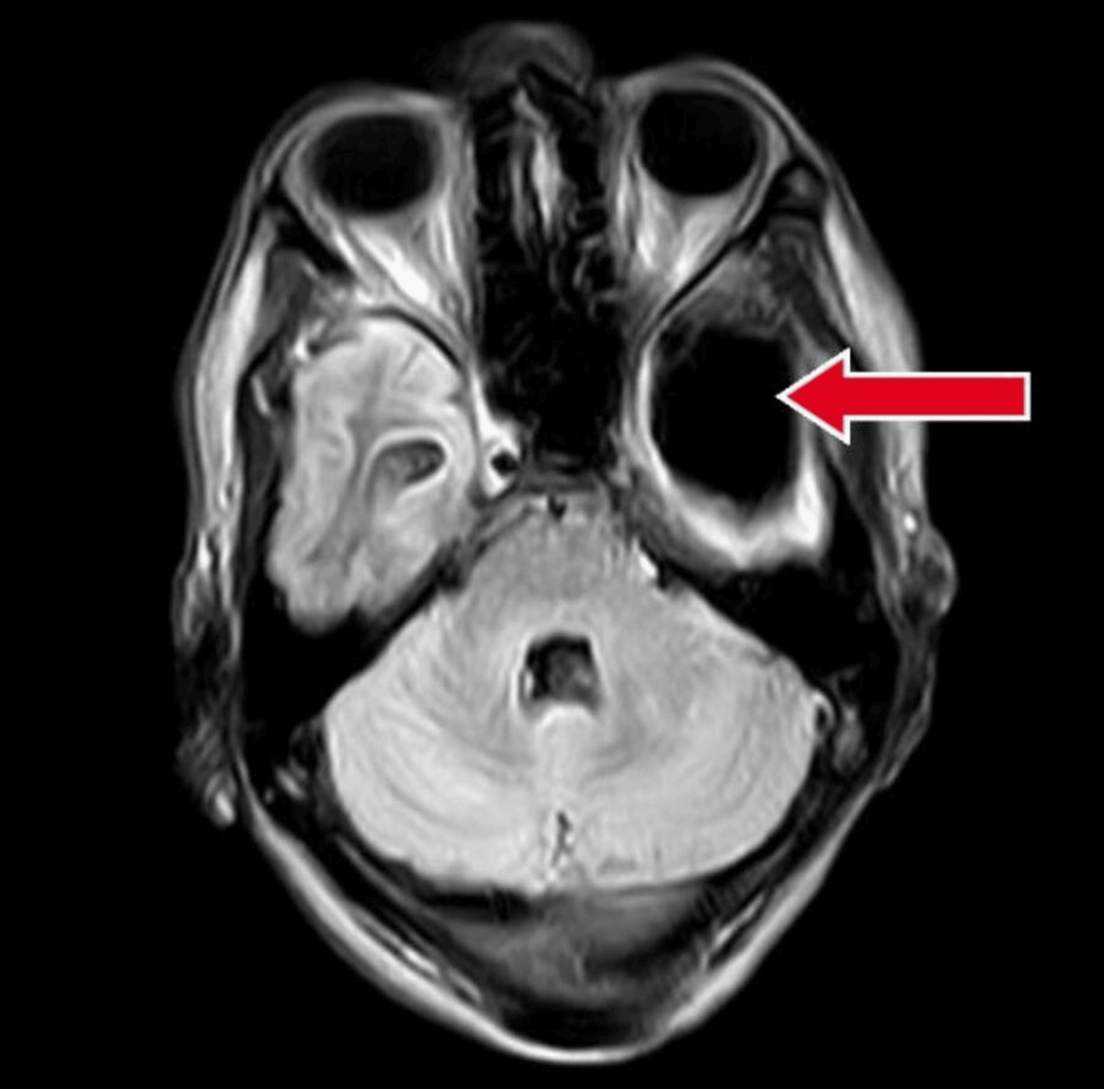
Contrast enhanced magnetic resonance imaging showing porencephalic cyst with red arrow.

**Figure 2:**
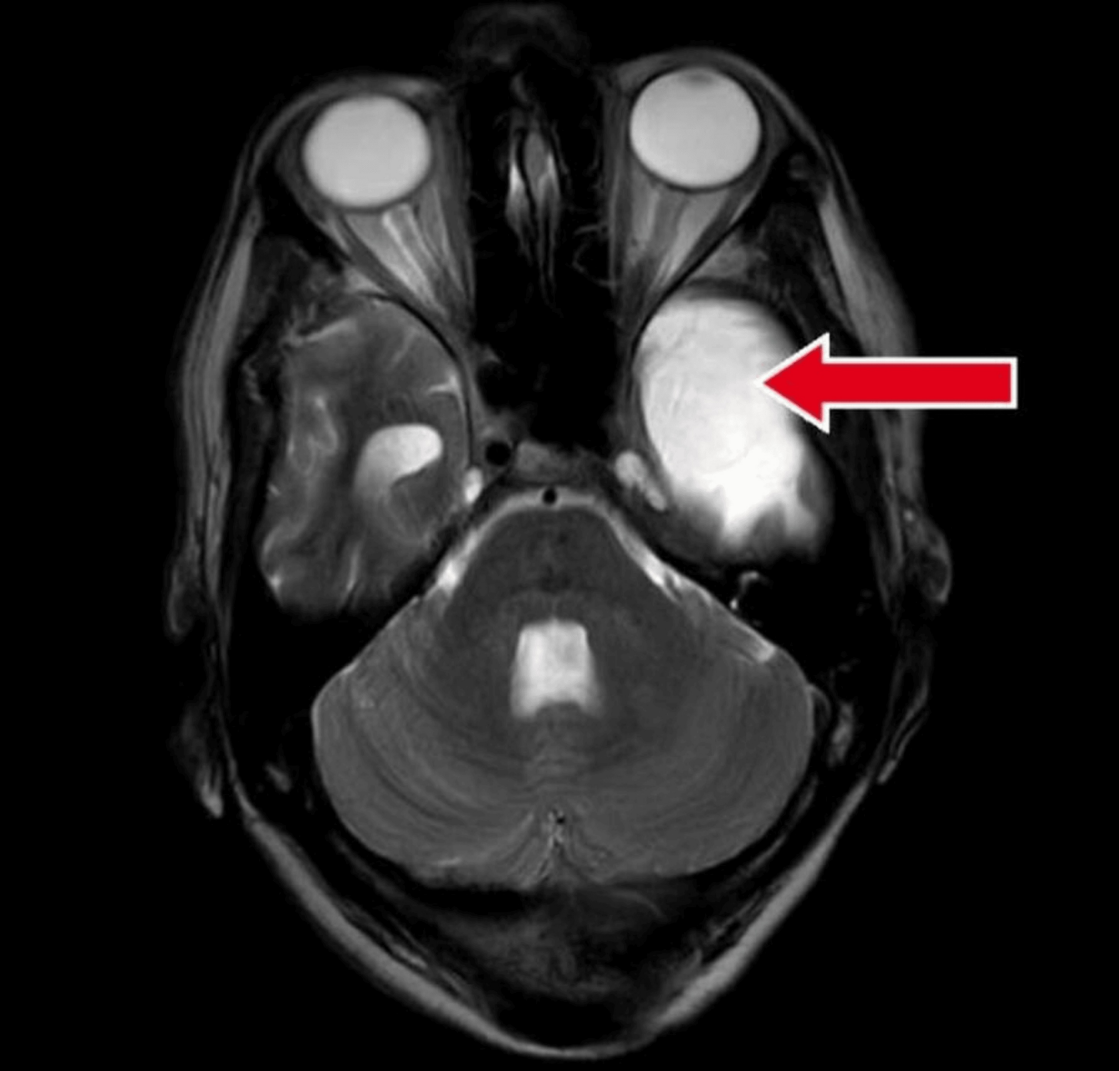
Contrast enhanced magnetic resonance imaging showing lesion in temporal lobe with red arrow.

**Figure 3:**
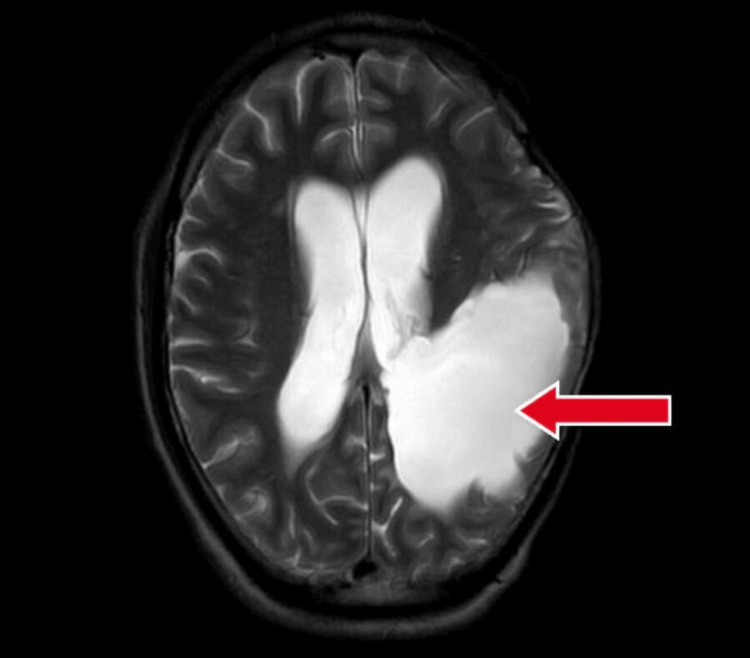
Contrast-enhanced magnetic resonance imaging showing lesion in temporal, parietal, and occipital lobes with red arrow.

**Figure 4:**
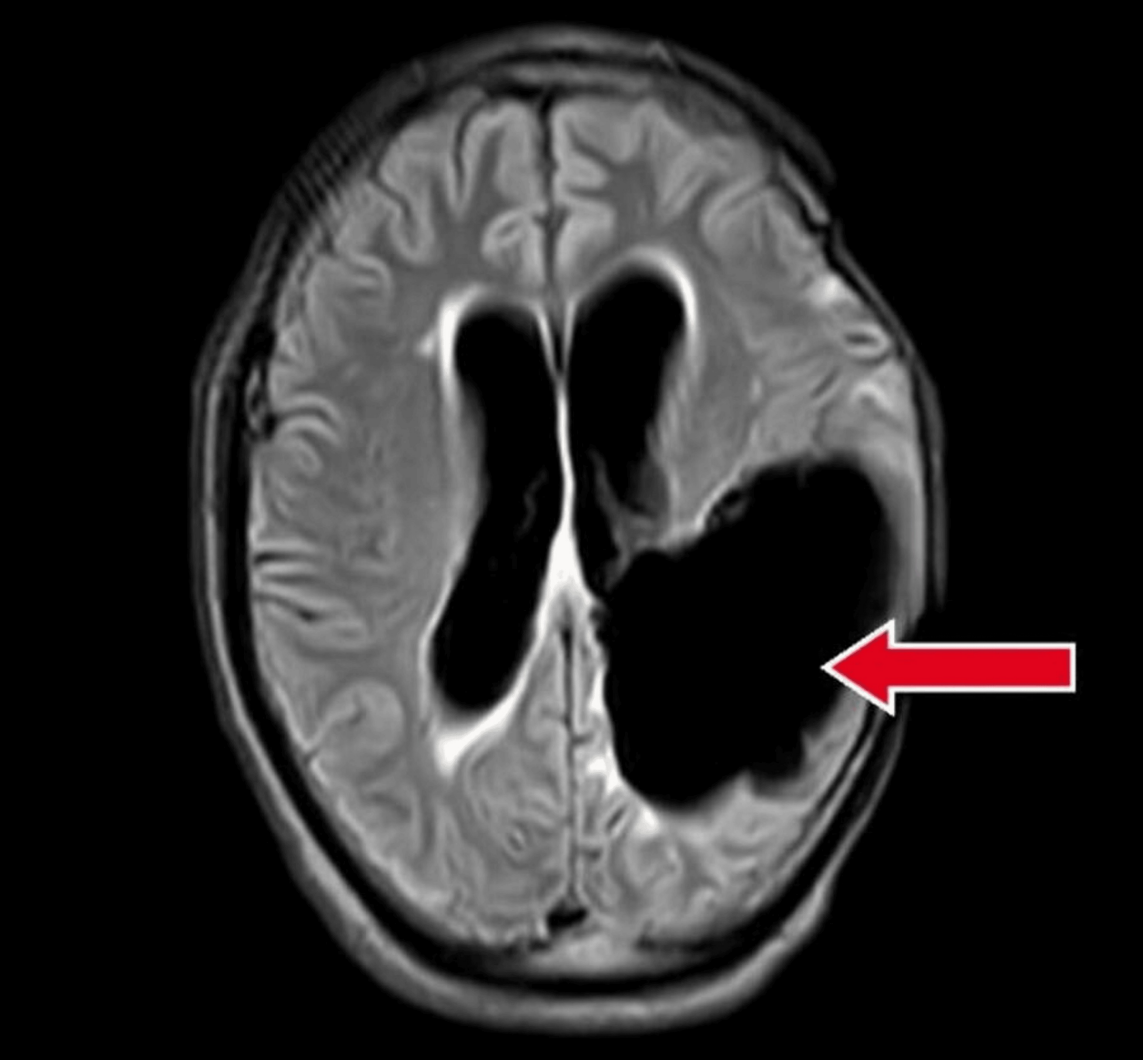
FLAIR magnetic resonance imaging showing lesion in the parietal and occipital lobes with red arrow. FLAIR: fluid-attenuated inversion recovery

Following presentation with current symptoms, a contrast-enhanced MRI brain was done which had an impression of a porencephalic cyst in the left parieto-temporo-occipital region along with hydrocephalus with residue of epidermoid cyst in the left parietal temporal region, as shown in Figures 1-4.

Routine investigations including complete blood count and liver and kidney function tests were within normal limits. A comprehensive physical and neurological examination of the patient revealed a decrease in power and muscle bulk in the right upper and lower limbs, while the cranial nerve examination remained within normal limits. The patient was further diagnosed with secondary psychotic syndrome, with delusions (6E61.1) according to the International Classification of Diseases 11th Revision (ICD-11) and was started on tab olanzapine which was gradually increased to 10mg in the night (one tablet/day) with which the patient was noted to have significant improvement in the symptoms of in the psychotic symptoms which includes delusion of persecution, delusion of reference, and made phenomenon. The patient was discharged on the above-mentioned dosage of medication and no further follow-up was done by the patient. The patient was advised no current intervention from the neurology.

## Discussion

Our case depicts a porencephalic cyst in the left parieto-temporo-occipital region along with the residue of the operated epidermoid cyst in the left parieto-temporal region. A cavity in the cerebral cortex with smooth walls called porencephaly connects directly to the ipsilateral lateral ventricle. Cerebrospinal fluid fills a porencephalic cyst, which is lined with white matter and has little mass impact. It happens after the white matter in the brain has necrotized. The patient in this instance showed spastic paresis in his right arm and leg along with motor impairment, which are clinical signs of porencephaly. This patient also experienced seizure-like symptoms with speech abnormality [7]. The presence of a porencephalic cyst in the frontal and temporal lobes may be linked to psychotic traits, or it may indicate that the patient's susceptibility to psychotic symptoms may have been induced by brain trauma [8]. To look into the aforementioned theory, we searched the scientific literature for other examples of porencephaly and insanity. In 2009, Pae et al. described a patient who experienced a psychotic episode that may have been brought on by numerous leukoencephalopathy and cortical porencephaly alterations [9]. The 30-year-old woman in question experienced visual hallucinations and persecutory thoughts. MRI findings included a significant porencephalic alteration in the right temporal lobe, bilateral occipital lobes and left basal ganglia, and several focal leukoencephalopathy changes in the left frontal lobe [9]. According to the research, schizencephaly (a rare birth defect of the brain characterized by abnormal clefts lined with grey matter that form the ependyma of the ventricles to the pia mater) is related to the presence of seizures, dizziness and episodes of loss of consciousness [9]. With an incidence of 3.5 per 100,000 live births, it is an uncommon event [10].

Although the exact cause of the symptoms is unknown, a variety of situations, including stressful life events, can cause symptoms in individuals and severe traumas [11-13]. There is a scarcity of research directly investigating the cause of porencephaly-inducing psychotic episodes. There are a few researches stating that the presence of a cyst in the temporal and parietal lobes can lead to cognitive impairment and disruption in emotional processing as the cyst affects the neuronal connections leading to inflammations of the particular site, which may lead to the presence of psychotic symptoms [8]. Treatment for porencephaly often focuses on symptom relief because there is no known way to stimulate brain growth in the areas of the brain that are absent. This may involve antiepileptic drugs, physical therapy, or the use of a shunt to drain extra cerebrospinal fluid. This case report contributes to the increasing body of research that suggests a potential link between porencephaly and psychosis, despite the paucity of available data. To validate this connection and investigate the underlying mechanisms, more investigation is required. Further research into this possible association may help guide future psychosis diagnosis and treatment plans.

## Conclusions

Research specifically examining the cause of porencephaly-induced psychotic episodes is scarce. A few studies have reported that the existence of a cyst in the parietal and temporal lobes can cause disruptions in emotional processing and cognitive impairment because the cyst affects the connections between neurons, causing inflammation at a specific location that can result in psychotic symptoms. In cases with porencephaly, treatment mostly consists of managing symptoms as there is no known means to promote brain growth in the absent parts of the brain. Antiepileptic medications, physical therapy, or the placement of a shunt to remove excess cerebral fluid may all be part of this. Despite the limited information available, this case report adds to the growing body of literature that raises the possibility of a connection between porencephaly and psychosis. Further research is needed to confirm this connection and look into the underlying mechanisms. Future diagnoses and treatment strategies for psychosis may benefit from more investigation into this potential correlation.
